# Progressive Cervical Mass Caused by a Laryngocele: A Case Report

**DOI:** 10.7759/cureus.97670

**Published:** 2025-11-24

**Authors:** Manar Almutiri, Mohamed Zahran

**Affiliations:** 1 General Surgery, Al Adan Hospital, Kuwait City, KWT; 2 Otolaryngology-Head and Neck Surgery, Alexandria University, Alexandria, EGY

**Keywords:** case report, cervical mass, laryngeal saccule, laryngocele, orl-hns, otolaryngology-head & neck surgeons, rare head and neck, surgical excision (se), swelling of neck

## Abstract

Laryngoceles are rare, benign dilations of the laryngeal saccule that may present with a wide range of symptoms or remain asymptomatic, making their diagnosis challenging. We present the case of a 24-year-old male soldier, a chronic smoker with a history of persistent cough, who presented with a painless, progressively enlarging swelling on the left side of his neck that increased with straining. He reported no dysphagia, dyspnea, or hoarseness, and both systemic and ENT examinations were unremarkable. Contrast-enhanced computed tomography (CECT) of the neck confirmed the diagnosis of an infected laryngocele. The patient underwent complete surgical excision via a transverse cervical incision under general anesthesia. Dissection was carefully performed through the sternocleidomastoid muscle, with complete removal of the cyst while preserving the superior laryngeal nerve. The postoperative course was uneventful, with normal voice and swallowing maintained. Histopathological analysis confirmed the diagnosis, and at one-year follow-up, the patient remained symptom-free with no evidence of recurrence. This case emphasizes the importance of considering a laryngocele in the differential diagnosis of painless neck swellings and demonstrates that surgical excision remains a safe and effective treatment with excellent long-term outcomes.

## Introduction

Laryngoceles, defined as an abnormal, air-filled dilation of the laryngeal saccule, also known as the saccule of the larynx or Hilton’s sac [1]. This condition is a rare condition, with an estimated incidence of only one case per 2.5 million people per year [2]. This small diverticulum originates from the laryngeal ventricle, which is a narrow horizontal slit between both the true and false vocal cords, communicating with the laryngeal lumen. Classified into internal, external, or combined based on their relation to the thyrohyoid membrane [1]. While this condition is mostly asymptomatic, symptoms such as cough, hoarseness, stridor, pain, sore throat, snoring, and a globus sensation might manifest in some patients. The term "laryngocele" is used when the lesion is symptomatic and palpable, confirmed by a laryngoscopy or by radiography, and extending above the superior margin of the thyroid ala [3]. This case is presented due to the rarity of laryngoceles in young adults, their potential to mimic other neck pathologies, and the important diagnostic and surgical lessons it provides.

## Case presentation

**Figure 1:**
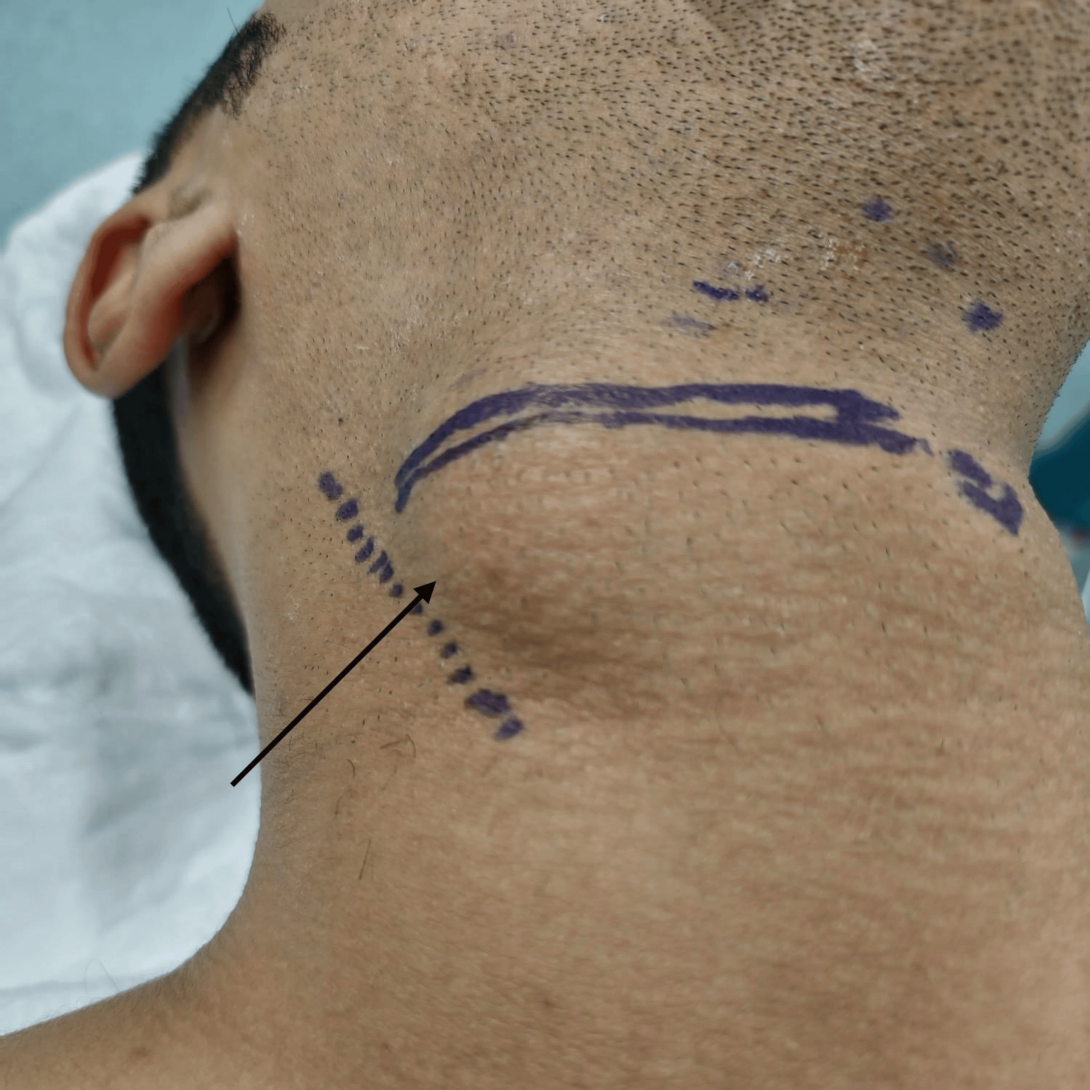
Preoperative photograph showing a right Level II neck swelling, with the planned transverse cervical incision marking. The Level II neck swelling classification is based on the Robbins Neck Level System [4] published by the American Head and Neck Society.

**Figure 2:**
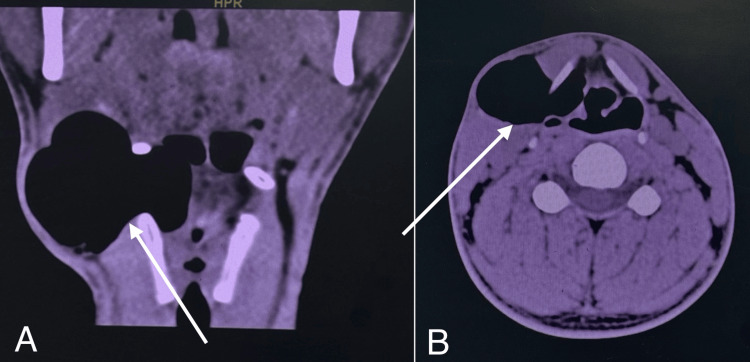
Non-contrast CT of the neck showing (A) coronal view with a right-sided air-filled swelling arising from the laryngeal saccule and extending through the thyrohyoid membrane, and (B) axial view confirming its lateral extension.

**Figure 3:**
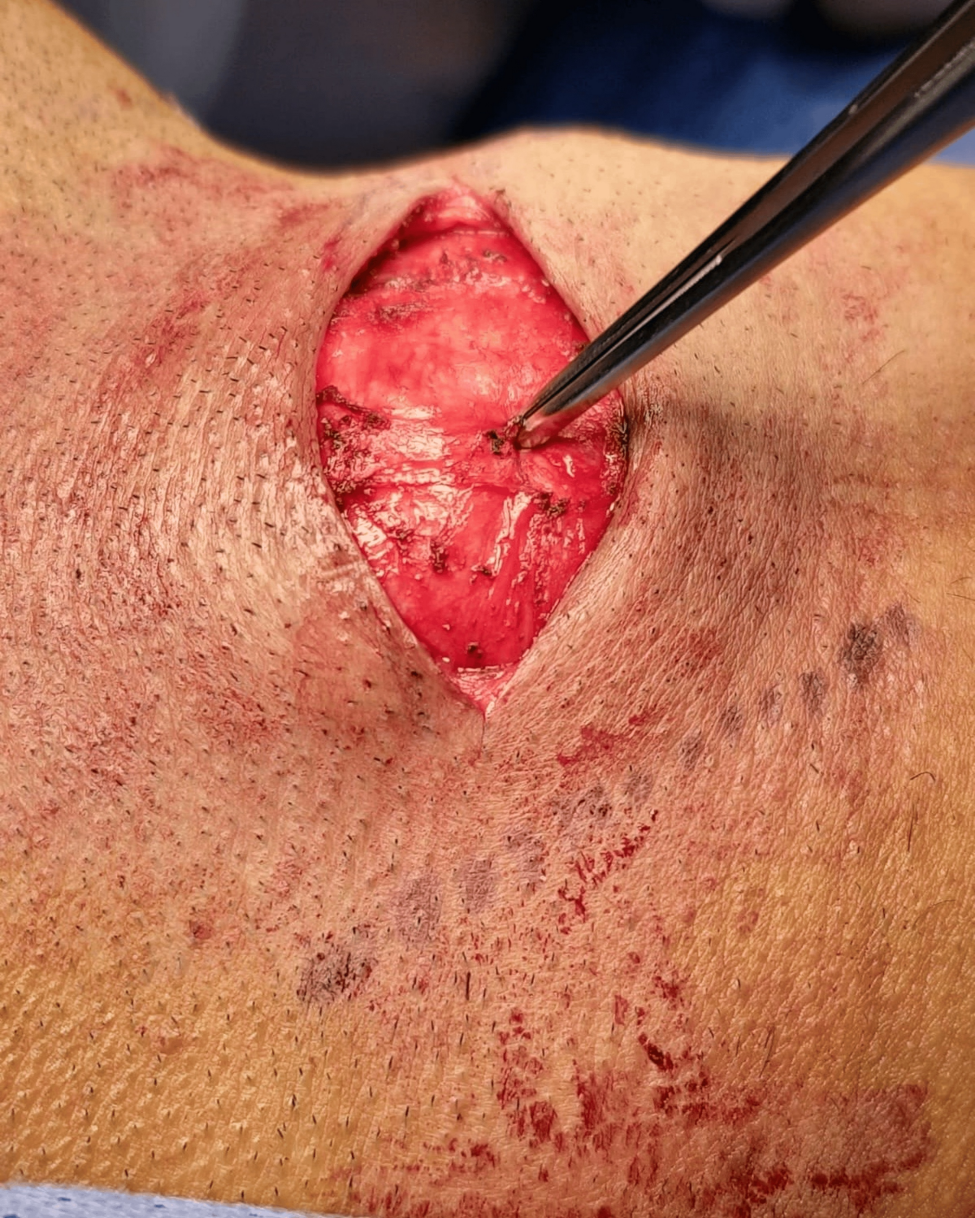
Transverse neck incision over the site of the laryngocele

**Figure 4:**
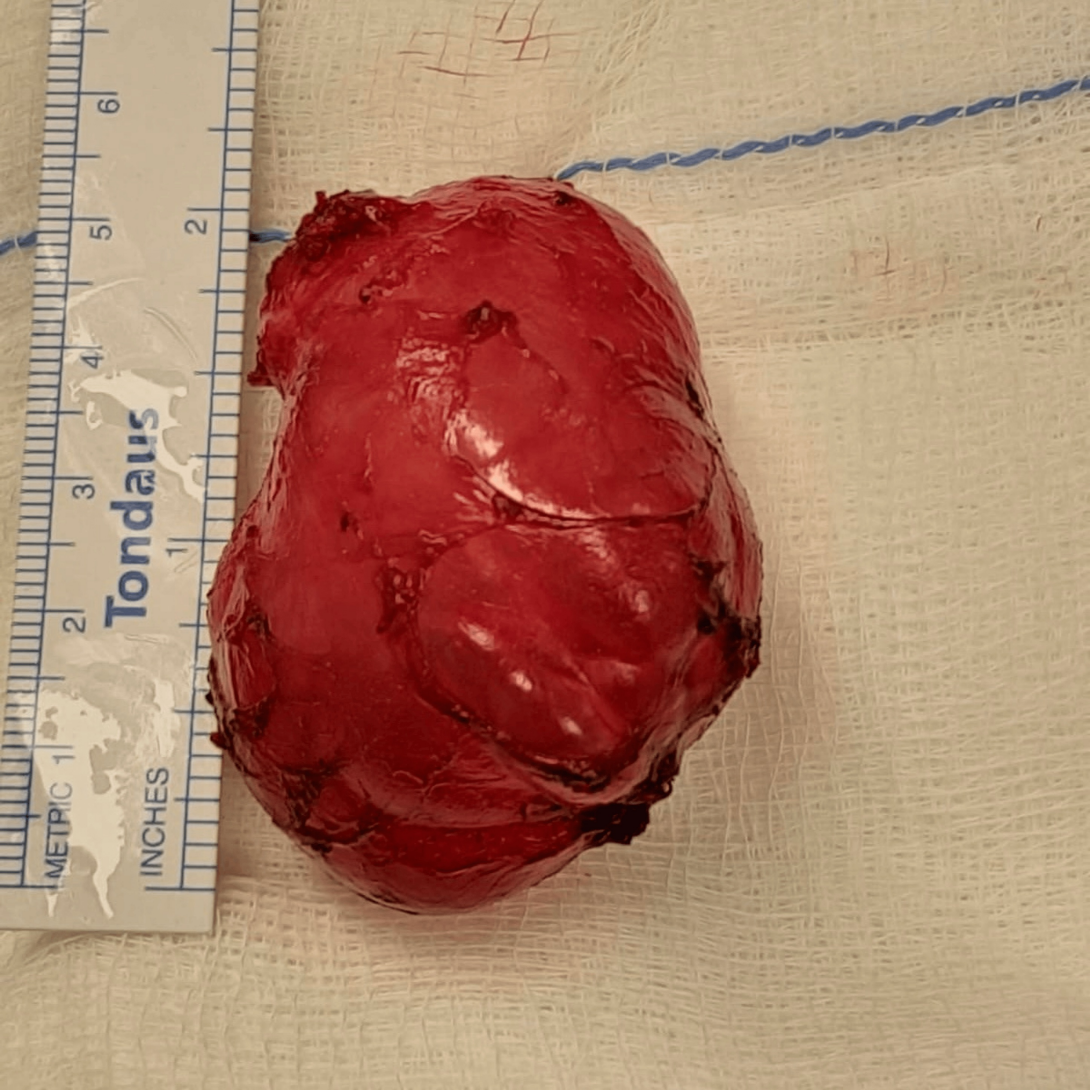
The laryngocele after complete excision

A 24-year-old male military soldier, a chronic smoker with a history of persistent cough, presented with painless, progressively enlarging swelling on the right side of his neck. The swelling increased in size with straining and coughing; the patient reported no dysphagia, dyspnea, or hoarseness. Past medical, surgical, and family histories were unremarkable. On clinical examination, the swelling was 3x5 cm in size, located on the lateral aspect of the neck, and was soft and compressible on palpation (Figure 1). Both systemic and ENT evaluations were otherwise normal. Flexible fiberoptic laryngoscopy revealed freely mobile vocal folds with a smooth bulge over the right false vocal fold, covered by intact mucosa. Contrast-enhanced computed tomography (CECT) of the neck was done, confirming the diagnosis of an infected laryngocele (Figure 2). The patient underwent surgical excision under general anesthesia with endotracheal intubation. A 3 cm transverse incision was made over the swelling, approximately two fingerbreadths below the lower border of the mandible (Figure 3). Subplatysmal flaps were elevated, and dissection was carried out through the sternocleidomastoid muscle, separating the cyst from the carotid sheath and superior laryngeal nerve. The cyst was traced to the laryngeal saccule and excised completely (Figure 4). Closure was achieved in two layers without the need for a drain. The postoperative course was uneventful with preservation of normal voice and swallowing. Histopathological examination confirmed the diagnosis of laryngocele. At the one-year follow-up, the patient remained symptom-free with no evidence of recurrence. 

## Discussion

The development of laryngoceles is thought to result from a combination of congenital predisposition and acquired factors. Acquired causes are often related to prolonged elevations in intralaryngeal pressure, as observed in glassblowers, wind instrument players, singers, public speakers, and individuals with chronic cough. Mechanical obstruction of the laryngeal ventricle opening by conditions such as carcinoma, chondroma, amyloidosis, or scarring can also create a valve effect that promotes saccule dilation [5]. When the neck of a laryngocele becomes obstructed, mucous secretions may accumulate, forming a laryngomucocele, which can further progress to a laryngopyocele if infected. Reported pathogens include *Staphylococcus aureus*, hemolytic Streptococcus B, *Escherichia coli*, and *Pseudomonas aeruginosa* [6]. Although uncommon, laryngoceles are more frequently encountered in men and typically present in the fifth or sixth decade of life [5]. Laryngopyoceles have also been described in association with laryngeal tumors and intravenous neck injections linked to drug abuse [6].

Diagnosing laryngoceles presents several challenges due to their varied clinical presentations and the need to distinguish them from other neck masses such as branchial cysts, saccular cysts, and laryngeal diverticula. Symptoms can be highly variable, ranging from hoarseness, snoring, and foreign body sensation to more severe issues like upper airway obstruction, dysphagia, and cough. This variability often necessitates a comprehensive investigation beyond initial clinical findings. A significant concern is the potential association between laryngoceles and laryngeal carcinoma, as approximately 10% of patients with laryngeal carcinoma have an accompanying laryngocele [7]. Therefore, imaging studies, particularly CT scanning of the neck, are crucial not only to determine the exact extension, anatomical relationships, and contents of the laryngocele but also to differentiate it from other conditions [8]. The findings from imaging are often confirmed through fine needle aspiration cytology [8]. Differential diagnoses include saccular cysts of the larynx, which also originate from the laryngeal saccule but differ in communication and content, and occult laryngeal tumors, which require careful exclusion through imaging and potentially histopathological analysis. Furthermore, an infected laryngocele, known as a laryngopyocele, must be differentiated from other inflammatory or infectious processes. The complex nature of these distinctions underscores the need for a multi-modal diagnostic approach to ensure accurate identification and appropriate management [7].

The management of laryngoceles has evolved over the past two decades, shifting from predominantly external approaches to more frequent endoscopic interventions, particularly for internal types. The choice of treatment depends on the size of the laryngocele, the symptoms it causes, and its anatomical classification as internal, external, or combined [7]. Small, asymptomatic laryngoceles may be managed conservatively with observation, while symptomatic cases presenting with hoarseness, dyspnea, dysphagia, or a palpable neck mass usually require surgical intervention. Traditionally, laryngoceles were excised using an external approach, but the advent of microlaryngoscopic surgery and the use of carbon dioxide (CO₂) lasers have made endoscopic management increasingly popular. For internal laryngoceles, microlaryngoscopy, particularly with a CO₂ laser, has become the primary therapeutic procedure, with 73.8% of internal cases in a reviewed dataset treated this way. This method is considered quick, precise, and safe, offering fewer complications and faster recovery compared to external excision. For larger or combined laryngoceles, the external approach remains the main therapeutic procedure, accounting for 86.2% of cases [5]. External techniques, such as the transthyrohyoid membrane approach, provide good exposure and a low recurrence rate but are associated with disadvantages like scarring, higher morbidity, and longer recovery times. While endoscopic techniques have also been explored for combined laryngoceles, and robotic surgery shows promise, their advantages still require further validation. In critical situations with risk of suffocation, acute resection or tracheotomy may be necessary. In cases where the laryngocele is secondary to an underlying laryngeal pathology, such as carcinoma, management must also address the primary disease [5].

## Conclusions

Laryngoceles, though rare, should always be considered when evaluating painless, progressive neck swellings, especially those that enlarge with straining. Early recognition and accurate diagnosis with imaging are essential to distinguish them from other laryngeal or cervical pathologies, including malignancy. Complete surgical excision remains a safe and effective treatment, offering excellent functional outcomes and minimal risk of recurrence. This case emphasizes the value of maintaining a broad differential diagnosis and highlights the importance of combining thorough clinical evaluation with appropriate imaging to guide timely management and ensure the best possible patient outcomes.
